# High alcohol-producing *Klebsiella pneumoniae* causes fatty liver disease through 2,3-butanediol fermentation pathway *in vivo*

**DOI:** 10.1080/19490976.2021.1979883

**Published:** 2021-10-10

**Authors:** Nan-Nan Li, Wei Li, Jun-Xia Feng, Wei-Wei Zhang, Rui Zhang, Shu-Heng Du, Shi-Yu Liu, Guan-Hua Xue, Chao Yan, Jing-Hua Cui, Han-Qing Zhao, Yan-Ling Feng, Lin Gan, Qun Zhang, Chen Chen, Di Liu, Jing Yuan

**Affiliations:** a Bacteriology Laboratory, Capital Institute of Pediatrics, Beijing, China; b Graduate School of Peking Union Medical College, Beijing, China; c Computational Virology Group, Center for Bacteria and Viruses Resources and Bioinformation, Wuhan Institute of Virology, Chinese Academy of Sciences, Wuhan, China; d University of Chinese Academy of Sciences, Beijing, China, Beijing, China; e CAS Key Laboratory of Pathogenic Microbiology and Immunology, Institute of Microbiology, Chinese Academy of Sciences, Beijing, China, Beijing, China; f Biomedical inovation center, Beijing Shijitan Hospital, Capital Medical University, Beijing, China

**Keywords:** HiAlc *kpn*, 2,3-butanediol fermentation pathway, FLD, ethanol, 2,3-butanediol

## Abstract

High alcohol-producing *Klebsiella pneumoniae* (HiAlc *Kpn*) in the gut microbiota had been demonstrated to be the causative agent of fatty liver disease (FLD). However, the catabolic pathways for alcohol production *in vivo* remain unclear. Here, we characterized the genome of HiAlc and medium alcohol-producing (MedAlc) *Kpn* and constructed an *adh* (an essential gene encoding alcohol dehydrogenase) knock-out HiAlc *Kpn* W14 strain (W14*Δadh*) using CRISPR-Cas9 system. Subsequently, we established the mouse model *via* gavage administration of HiAlc *Kpn* W14 and W14 *Δadh* strains, respectively. Proteome and metabolome analysis showed that 10 proteins and six major metabolites involved in the 2,3-butanediol fermentation pathway exhibited at least a three-fold change or greater during intestinal growth. Compared with HiAlc *Kpn* W14-fed mice, W14*Δadh*-fed mice with weak alcohol-producing ability did not show apparent pathological changes at 4 weeks, although some steatotic hepatocytes were observed at 12 weeks. Our data demonstrated that carbohydrate substances are catabolized to produce alcohol and 2,3-butanediol *via* the 2,3-butanediol fermentation pathway in HiAlc *Kpn*, which could be a promising clinical diagnostic marker. The production of high amounts of endogenous alcohol is responsible for the observed steatosis effects in hepatocytes *in vivo*.

## Introduction

*K. pneumoniae* is responsible for opportunistic community-acquired infections such as pneumonia, urinary tract infection, and pyogenic liver abscess, and presents an urgent threat in hospital settings due to the emergence of multidrug-resistant and hypervirulent strains. The emergence of antibiotic-resistant strains of *K. pneumoniae* worldwide has become a cause of concern with extended spectrum β-lactamases and carbapenemase-producing strains being isolated with an increasing frequency.^1^ Furthermore, the incidence of bacterial pneumonia has increased in alcoholic patients, and *K. pneumoniae* is a cause of severe pneumonia in alcoholics in Africa and Asia.^2,3^ Although substantial research has been carried out on *K. pneumonia*, our previous study was the first to connect its high alcohol-producing characteristics with FLD.^4^

Microbial production of alcohols such as ethanol, propanol, butanol, and other short-chain alcohols has been investigated by many researchers.^5^ In our previous work, we found that HiAlc *Kpn* is associated with up to 60% of individuals with FLD in a Chinese cohort, and we proposed that, in at least some cases of FLD, the alteration in the gut microbiome drove the condition because of the excess endogenous alcohol production.^4^ Therefore, we named this kind of disease as endogenous alcohol fatty liver disease (endo-AFLD). FLD, a precursor of cirrhosis and hepatocellular carcinoma, is the most common chronic liver disease worldwide. However, the underlying fermentation pathway of ethanol production *in vivo* by the HiAlc *Kpn* as a contributing player in microbiota-induced FLD/steatohepatitis is still unclear. Furthermore, how the metabolic profiling of HiAlc *Kpn in vivo* affects the FLD remains unknown. In our present work, the genomic characteristics of HiAlc *Kpn* and MedAlc *Kpn* isolated from an auto-brewery syndrome (ABS)/nonalcoholic steatohepatitis (NASH) patient were analyzed. In addition, an endogenous alcohol-producing pathway in HiAlc *Kpn* was identified and confirmed by *in vivo* and *in vitro* experiments. The metabolic alterations associated with alcohol induction during the development of hepatic steatosis have also been demonstrated using a murine model, which may offer a target for intervention or a marker for disease. In general, this study augmented the current knowledge and clarified the link between alcohol-producing *Kpn* and FLD.

## Results

### Bacterial characteristics and genomic analysis of HiAlc and MedAlc *Kpn* strains

**Figure 1.**
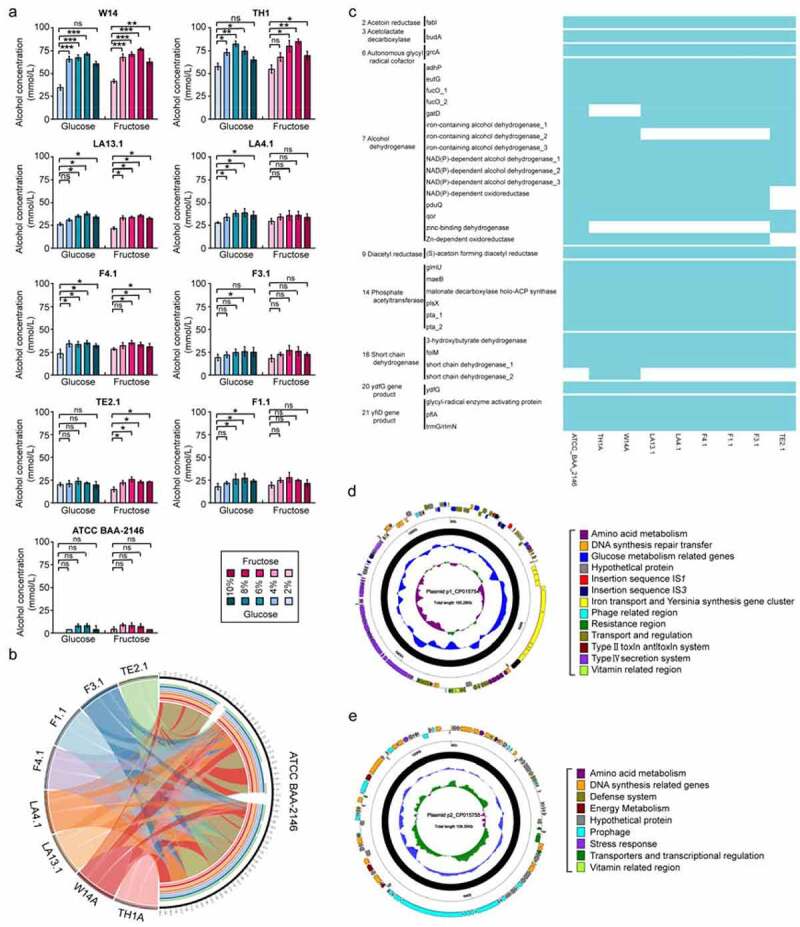
The alcohol-producing abilities and the genomes of HiAlc and MedAlc *Kpn* isolates. (a) The alcohol concentration of HiAlc and MedAlc *Kpn* cells in YPD medium with a single carbon source (fructose or glucose) (n = 3). Values denote means ± SD. *p* values were calculated by two-way ANOVA test (ns = not significant). (b) The alcohol-producing genes among the genomes of nine *Kpn* strains (ATCC BAA-2146, W14, TH1, LA13.1, LA4.1, F4.1, F1.1, F3.1, and TE2.1). Each ribbon represents the homologous syntenic region between the *Kpn* strain and the reference strain ATCC BAA-2146. Each color represents a *Kpn* strain. (c) Sequence comparison among nine strains based on the 34 genes related to alcohol-producing metabolic pathways. Comparison of the alcohol-producing genes among the genomes of nine *Kpn* strains (ATCC BAA-2146, W14, TH1, LA13.1, LA4.1, F4.1, F1.1, F3.1, and TE2.1). The light blue blocks indicate the presence of the genes listed on the left, while the blank blocks indicate the absence of the related genes. (d, e) Schematic maps of p1_CP015754 (d) and p2_CP015755 (e) genes are denoted by arrows and are colored based on gene function classification. The innermost circle presents GC-skew [(g-c)/(G + C)], with a window size of 500 bp and a step size of 20 bp. The blue circle presents GC content, inward means lower than average GC content, and outward means higher than average GC content. the backbone regions are highlighted in black. The next-to-innermost circle presents GC content. The outermost circle presents the distribution of genes

The presence and content of HiAlc or MedAlc *Kpn* in the fecal flora were significantly associated with FLD.^4^ As described in our previous work, we isolated a total of eight non-repeated alcohol-tolerant strains of *K. pneumoniae* from the clinical fecal samples from an ABS/NASH patient. In this work, according to the alcohol-producing ability of *Kpn*, we divided the strains into HiAlc *Kpn* (≥ 40 mmol/L, W14, TH1; 30–40 mmol/L, LA13.1, LA4.1, and F4.1) and MedAlc *Kpn* (20–30 mmol/L, F1.1, F3.1, and TE2.1) (Figure 1a). Interestingly, we found that the growth speed of HiAlc and MedAlc *Kpn* strains was more rapid than that of the standard strain ATCC BAA-2146 with low alcohol-producing ability and reached exponential phase within 2 h, in particular, under aerobic conditions, as determined by bacterial growth curves. This is likely related to competitiveness with other microbiota during *in vivo* growth (Figure S1). Moreover, all *K. pneumoniae* isolates typically expressed O antigen and K antigen on its cell surface. They belonged to K30 serotype and were classified as different MLST types including ST323 (LA13.1, LA4.1, F4.1, F1.1, and F3.1), ST1536 (W14 and TH1), and a new ST type (TE2.1). Besides, the strains possessed five types of genes encoding potential virulence-related factors, including *Kfu*BC, *ure*A, *wab*G, *uge*, and *ybt*A.

To investigate the roles of genomic heterogeneity and capsular serotypes in the pathogenesis of *K. pneumoniae* strains, we sequenced the genomes of these strains were sequenced (Figure 1b, c). Sequencing results showed that the genome sizes of the eight isolated strains ranged from 5,296,799 bp to 5,584,287 bp, encoding 5053–5911 open reading frames (ORFs), along with 0 to 2 plasmids. The length of the plasmids ranged from 104,656bp to 1,893,830 bp (Table S1). In these strains, approximately 73%/ 97% of the proteins had COG/KEGG annotations, except for strain TE2.1, which had only 51.28%/74.73% of annotations, respectively (Table S1). We focused on the genes involved in the alcohol-producing pathway. The gene sequences were selected for alignment using CLC Genomics Workbench 11. As shown in Figure 1c, thirty-four genes were listed according to their functional annotations and protein sequence similarity, and the numbers of genes were counted in each strain. Compared with ATCC BAA-2146, the HiAlc *Kpn* strains W14 and TH1 possessed short chain dehydrogenase 2 but lacked the *gat*D gene and zinc-binding dehydrogenase. The HiAlc *Kpn* strains LA13.1, LA4.1, and F4.1, and the MedAlc *Kpn* strains F1.1 and F3.1 lacked iron-containing alcohol dehydrogenase 2. The MedAlc *Kpn* strain TE2.1 lacked NADP-dependent oxidoreductase, *pdu*Q, and Zn-dependent oxidoreductase.

For HiAlc *Kpn* strains with the highest alcohol-producing ability such as W14 and TH1, the assembled genomes of HiAlc *Kpn* were so similar that two Klenow fragments underwent transversion, consisting of a single circular chromosome of 5,220,857 bp and 5,220,846 bp with an average GC content of 57.12%. The 5,212 predicted ORFs covered 95% of the genome (Table S1). We compared the HiAlc *Kpn* strains with other *K. pneumoniae* strain sequences deposited in GenBank database, the results revealed that most of the different genes were located in four regions according to the BLAST Ring Image Generator (BRIG). What’s more, of the 333 genes specific to these strains, three large-region sequences were predicted to be elements of prophage by PHASTER software and type-VI secretory system elements, which contribute to bacterial competition, cell invasion, type-I fimbriae expression, and colonization *in vivo*.^6^

It would be worth emphasizing that a novel plasmid of 165,276 bp possessed a homologous segment of ~3000 bp encoded genes with 99% identity to *Yersinia pestis* 3770, *Escherichia coli* UMN026, and *K. pneumoniae* (from position 29121 to 29194) was found (Figure 1d). Four interesting functional regions were detected by nucleotide sequence analysis. Eight genes were found to be related to carbohydrate metabolism and alcohol-production, which included short chain dehydrogenase, NADPH-dependent oxidoreductase, Family 1 glycosylhydrolase, SDR family oxidoreductase, phosphotransferase, and GNAT family N-acetyltransferase. Furthermore, the presence of insertion sequences and IS1 and IS3 integrons implied the transfer and integration of some genes. Notably, a 29,911bp fragment was similar to the high-pathogenicity island of *Yersinia pestis* and *Y. pseudotuberculosis*, which included the synthesis of a yersiniabactin siderophore system and played an important role in the pathogenesis of *K. pneumoniae* infection.^7^ Studies have assayed the distribution of siderophores among *K. pneumoniae* clinical isolates and have found that nearly all of them produce enterobactin, whereas a much smaller percentage produce either aerobactin or yersiniabactin.^8^ In addition, we further demonstrated the production of yersiniabactin in HiAlc *Kpn* strains by cross-feeding assays, which showed that culture supernatants of HiAlc *Kpn* strains promoted the growth of an indicator strain (*Yersinia enterocolitica* strain 201) under iron-deficient conditions. Furthermore, a 47,878 bp genomic island encoded a potentially functional type-F conjugative DNA transfer system, which was a paradigm for bacterial conjugation, transfer and represents a subset of the type IV secretion system (T4SS). The F sex factor of *E. coli* is well known as a paradigm for bacterial conjugation and its transfer (tra) region represented a subset of the T4SS family. The F *tra* region encodes eight of the 10 highly conserved (core) gene products of T4SS including TraA (pilin), TraB, TraK (secretin-like), TraV (lipoprotein), TraC (NTPase), TraE, TraL, and TraG (N-terminal region), and a cluster of genes encoding proteins essential for F conjugation (TraF, -H, -U, -W, the C-terminal region of TraG, and TrbC) that are hallmarks of the F-like T4SS. T4SS has also been found to secrete virulence factor proteins directly into host cells, as well as take up DNA from the medium during natural transformation, suggesting the versatility of this macromolecular secretion apparatus.^9^ Horizontal gene transfer contributes substantially to genomic heterogeneity among bacteria. The exchange of DNA plays a critical role in the evolution of bacteria and facilitates the rapid adaptation of bacteria to environmental alterations. At present, bacterial conjugation provides a model system for studying bacterial signaling as the nature of the elusive mating signal remains unknown. It was anticipated that an external cue, possibly involving contact between the donor and recipient, is transferred *via* the pilus, through membrane-associated T4SS and coupling protein to the cytoplasmic relaxosome. This process appears to involve pilus retraction, which is a poorly understood phenomenon to date. Several T4SS components contain features of signaling molecules. This signal could then trigger events that resemble phage infection and injection of DNA or the injection of proteins in a contact-mediated manner. The function of a plasmid carrying T4SS in HiAlc *Kpn* W14 and TH1 required further study.

Another plasmid of 109,349 bp shared conserved sequences with five previously reported plasmids namely pPMK1-B, pKPHS1, and pKO_JKo3_2 in *K. pneumoniae* or *K. oxytoca*, pCAV1741-110 in *Citrobacter freundii* strain CAV1741, and pMT in *Y. pestis* 1522 (Figure 1e). Detailed information and sequences for the pipeline have been deposited in the National Center for Biotechnology Information genome database (accession numbers of NZ_CP015753.1 for W14 and NZ_CP016159.1 for TH1; www.ncbi. nlm.nih.gov/genome/annotation_ prok/).

The genes encoding all of the enzymes of the Embden-Meyerhof-Parnas pathway (EMP), hexose monophosphoric acid pathway (HMP), Entner-Doudoroff (ED) pathway, and Tricarboxylic acid cycle (TCA) pathway were present in *K. pneumoniae* W14 and TH1. Analyses of the genome sequences revealed the determinants of hexose-metabolizing enzymes such as invertase, levansucrase, glucokinase, glucose-6-phosphate isomerase, and glucose-fructose oxidoreductase. These enzymes would enable *K. pneumoniae* to use sucrose, fructose, and glucose (as well as probably glycerol, mannose, raffinose, and sorbitol), then, convert acetyl-coA to acetaldehyde using MhpF and AdhE, and finally, produce alcohol through alcohol dehydrogenases (ADHs). More than 12 highly specific ADHs could catalyze the conversion of acetaldehyde to ethanol. Furthermore, most of these ORFs were also found to be actively transcribed in association with ethanol production by *K. pneumoniae* W14 and TH1. These results strongly suggested that the rapid production and high yield of ethanol could probably be attributed to the presence of 12 ADHs and pyruvate decarboxylase (GL003732 and GL001278, thiamine pyrophosphate protein TPP-binding domain protein [EC:4.1.1.74]), an enzyme not frequently observed in bacteria.

### Identification of the alcohol-producing pathway in HiAlc Kpn

To investigate the genes expression and further determine the key fermentation pathway underlying alcohol production, we performed comparative proteomic and metabolic analyses in murine feces *in vitro* and i*n vivo* during cultivation in rabbits.

**Figure 2.**
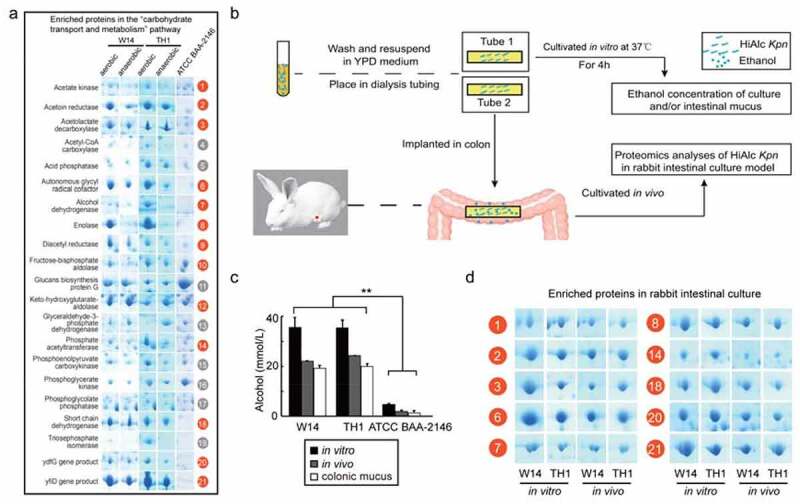
Differential proteomics analysis of *K. pneumoniae* W14 and TH1 under aerobic and anaerobic conditions. (a) Significant proteins in HiAlc *Kpn* W14 and TH1 cells enriched under aerobic and anaerobic conditions, showing 20 up-regulated proteins and one down-regulated protein, which are related to carbohydrate transport and metabolism, especially for the proteins related to the 2,3-butanediol fermentation pathway. *Kpn* ATCC BAA-2146 was used as a control. (n = 3, Data are expressed for at least three independent experiments). (b) The models for rabbit intestinal culture and proteomics analysis of HiAlc *Kpn in vivo* and *in vitro*. (c) Alcohol concentration of the intestinal mucus after culturing with HiAlc *Kpn* W14 and TH1 *in vivo* and *in vitro* for 4 h. The standard strain ATCC BAA-2146 was used as a control. The experiments were repeated at least three times (n = 3 rabbits/group/ experiment), data are denoted as means ± SD. The *p* values were calculated by two-way ANOVA test. (d) Ten up-regulated proteins in HiAlc *Kpn* W14 and TH1 were found to be associated with ethanol production through the 2,3-butanediol fermentation pathway. The numbers with red circles indicate the identified proteins. The experiments were repeated at least three times (n = 3 rabbits/group/experiment)

Considering given the differences in the alcohol production of HiAlc *Kpn* under aerobic (63.2 for W14 and 60.8 mmol/L for TH1, respectively) and anaerobic conditions (36.7 for W14 and 31.2 mmol/L for TH1, respectively),^4^ we separated and identified the proteins that were expressed by *K. pneumoniae* W14 and TH1 cultivated under these different conditions. The expression levels of the cultures grown under anaerobic conditions were very similar to those grown under aerobic conditions, and many of the landmark spots had counterparts. A total of 66 protein entries, which exhibited at least a three-fold change or greater, comprising 59 up-regulated and seven down-regulated proteins (Table S2), were identified by matrix-assisted laser desorption/ ionization-time of flight/time of flight-tandem mass spectrometry and/or electrospray ionization-tandem mass spectrometry during aerobic growth. Notably, 21 proteins (accounting for 32%) were enriched in the carbohydrate transport and metabolism pathway (Figure 2a). These identified proteins were related to metabolism, stress, and translation. We detected these 21 proteins: acetate kinase, acetoin reductase, acetolactate decarboxylase, acetyl-CoA carboxylase, acid phosphatase, autonomous glycyl radical cofactor, ADH, enolase, diacetyl reductase, fructose-bisphosphate aldolase, glucans biosynthesis protein G, keto-hydroxyglutarate aldolase, glyceraldehyde-3-phosphate dehydrogenase, phosphate acetyltransferase, phosphoenolpyruvate carboxykinase, phosphoglycerate kinase, phosphoglycolate phosphatase, short chain dehydrogenase, triosephosphate isomerase, *yfi*G gene product, and *yfi*D gene product. In particular, the proteins involved in the 2,3-butanediol fermentation pathway including ADH, acetate kinase, acetolactate decarboxylase, diacetyl reductase, and acetoin reductase, and the proteins related to pyruvate formate-lyase flux such as the *yfi*D gene product, *pfl*B gene product, and autonomous glycyl radical cofactor were uniquely highly expressed in HiAlc *Kpn* but were not up-regulated in the standard strain ATCC BAA-2146, suggesting that carbohydrate substances are catabolized to produce ethanol and 2,3-butanediol *via* the 2,3-butanediol fermentation pathway (Figure 2a).

We further analyzed HiAlc *Kpn* proteomics within the intestine using a rabbit intestinal culture model (Figure 2b), which we used for *in vivo* bacterial analysis.^4^ A higher alcohol concentration (~20 mmol/L) was detected in the colonic mucus after four hours cultivation *in vivo* (Figure 2c). In a rabbit intestinal culture model, 10 of the 21 proteins mentioned above were identified, including enzymes involved in the 2,3-butanediol fermentation pathway (Figure 2d). These results suggested that the *in vitro* ability of HiAlc *Kpn* to produce alcohol may reflect the status of such bacteria *in vivo*.

### HiAlc *Kpn* produces alcohol by 2,3- butanediol pathway in vivo

**Figure 3.**
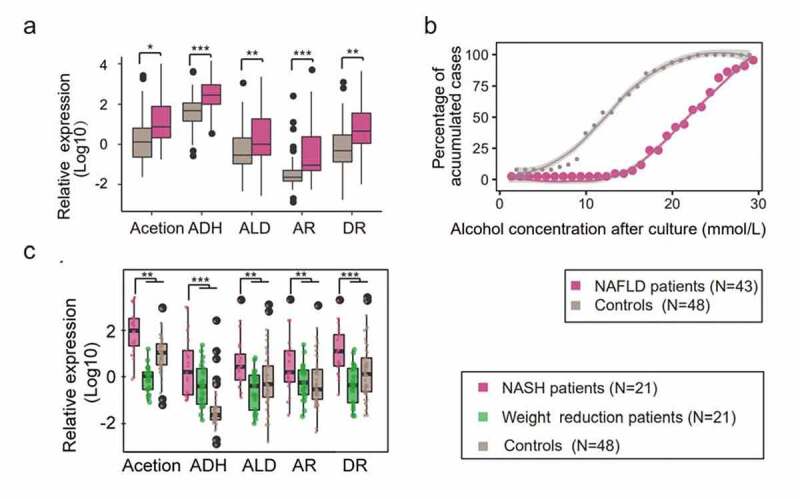
Confirmed 2,3-butanediol pathways of *Kpn* in FLD patients. (a) Evaluation of the alcohol- producing genes in patients with FLD (n = 43), including NAFL (n = 11) and NASH (n = 32), and the healthy individuals (n = 48) by RT-PCR analysis. (b) FLD patients (n = 43) had a higher chance of containing HiAlc *Kpn* compared with the controls (n = 48). The LOESS smoothing method was used to regress the relation between accumulated cases and *Kpn* alcohol producing ability with gray shadows. (c) Alcohol producing genes in fermented fecal samples of NASH patients (carmine box), weight-loss NASH patients (green box), and controls (brown box). In the box plot, (a-c) the center line indicates the median; box outlines show 25th and 75th percentiles, and whiskers indicate 1.5× the interquartile range. Extreme values are shown separately (black dot). Values are expressed as mean ± SD

To further validate 2,3-butanediol pathways *in vivo* of HiAlc *Kpns*, we analyzed the expression of genes associated pathways in patients with FLD (n = 43) and control subjects without FLD symptoms (n = 48). Results showed that the key enzyme genes associated with alcohol producing pathways were expressed at higher levels in FLD patients compared with those in healthy individuals (Figure 3a). In our cohort, 60% FLD patients carried HiAlc and MedAlc *Kpn* (alcohol-producing concentration ≥ 20 mmol/L), while that was only 6.25% in controls (Figure 3b). Also, more expression of the key enzyme genes was identified in patients with FLD, which was in accordance with the increasing abundance of HiAlc *Kpn* and alcohol concentrations of the fecal culture.

To investigate whether the fermentation of ethanol *in vivo* and the presence of HiAlc *Kpn* was associated with clinical characteristics of the patients, we further followed-up 21 of 32 NASH patients from our cohort after 6–9 months, who lost weight for averaged 20 ± 4.2 kg and have recovered from FLD because examinations of their hepatic steatosis were within normal ranges. In these patients, HiAlc or MedAlc *Kpn* had ever been isolated from their fecal samples. Generally, these patients were well controlled by diet, physical practice and cured. The data revealed that key enzyme genes associated with alcohol- producing pathways were expressed at lower levels in FLD patients followed-up compared with those of controls (Figure 3c). Taken together, these results indicate that the HiAlc *Kpn* as a potential cause of FLD could induce the production of endogenous ethanol via 2,3- butanediol pathways.

### Increased fecal metabolites in FLD in vivo

**Figure 4.**
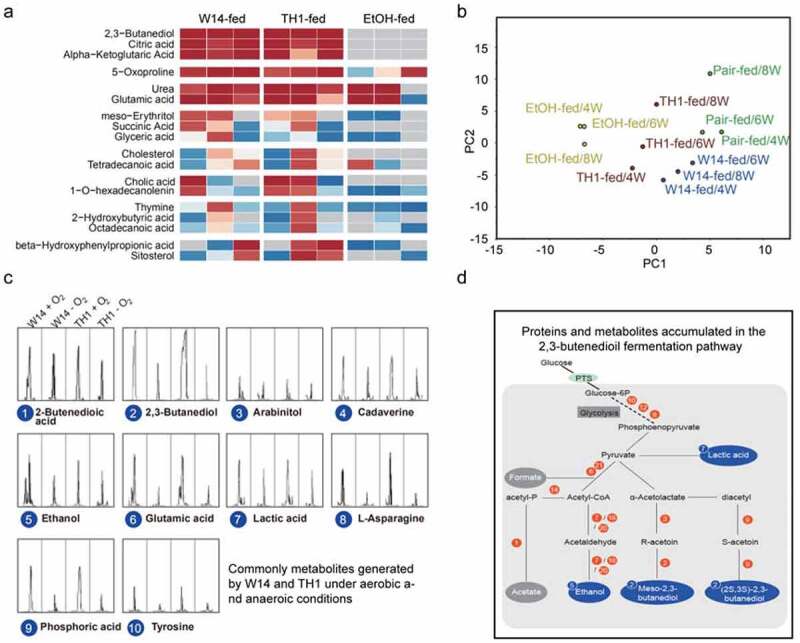
Metabolomic profiling of fecal samples from mice with FLD induced by HiAlc *Kpn* feeding and that of cells cultivated under aerobic and anaerobic conditions, with GC-MS peaks indicating volatile organic compounds. (a) Comparative metabolomic analysis of mice with HiAlc *Kpn*-induced FLD, showing the ratios of metabolite intensity in HiAlc *Kpn*-fed and EtOH-fed mice (against pair-fed), and the increased level at different stages of metabolites grouped according to the pattern (HiAlc *Kpn*-fed vs. EtOH-fed). (b) Principal component analysis of FLD mice induced by HiAlc *Kpn*-fed, ethonal-fed, and pair-fed at 4, 6, and 8 weeks. (c) Common metabolites derived from cultured HiAlc *Kpn* W14 and TH1 strains (peak intensity ≥ 3.8e+006). (d) Proposed central metabolism of the 2,3-butanediol fermentation pathway in HiAlc *Kpn*, in which end products and intermediates are presented in bold. The enzymes identified in the pathway are shown with red numbers, and the metabolites identified by GC-MS are shown in blue circles. Numbers with red or blue circles are presented according to the proteins or metabolites identified in Figure 2a, Figure 2d, and Figure 4d. Two-way ANOVA presented significant differential expression among these groups (n = 3). Data are expressed as mean ± SD of ≥ 3 independent experiments

To explore global metabolic alterations associated with alcohol induced during the development of hepatic steatosis, fecal metabolites in HiAlc *Kpn*-fed FLD mice and control mice at 4, 6, and 8 weeks were acquired as volatile organic compounds and analyzed by GC-MS (Figure S2, Table S3). Analysis of the metabolomic profiles in combination with principal component analysis revealed significant differences in metabolites among the HiAlc *Kpn*-fed, ethanol-fed, and pair-fed groups (Figure 4a, b). The ratio of metabolite peak intensity of the HiAlc *Kpn*-fed or ethanol-fed mice to the pair-fed mice was thus used as an indicator. A total of 18 main metabolites were significantly increased in HiAlc *Kpn*-fed or ethanol-fed mice, including urea, alcohols, sugars, amino acids, and acids (Figure 4a). Further analysis showed that these metabolites were all related to alcoholism, fatty metabolism, nitrogen metabolism, primary bile acid biosynthesis, butanoate metabolism, retrograde endocannabinoid signaling, and key pathways associated with alcohol production. Of special interest, we noticed that six main metabolites showed continuously elevated levels in HiAlc *Kpn*-fed mice, but not in ethanol-fed mice (Figure 4a). Three metabolites, including 2,3-butanediol (with the highest peak intensity), citric acid, and alpha-ketoglutaric acid, had higher concentrations in HiAlc *Kpn*-fed mice, but failed to be detected in ethanol-fed mice, suggesting that HiAlc *Kpn* could produce these metabolites as well as ethanol *in vivo*.

In addition, we compared metabolites that were detected under both aerobic and anaerobic conditions and presented the differences via GC-MS. We confirmed that 10 metabolites, including 2,3-butanediol, ethanol, lactic acid ethanolamine, urea, and other metabolomes, were present with an intensity above 3.8e+006 in the supernatant of *K. pneumoniae* strains W14 and TH1 grown for 12 h (Figure 4c). This was in accordance with the abundant metabolites identified in the fecal samples of HiAlc *Kpn*-fed mice.

By scanning all potential alcohol-producing pathways in bacteria, we found that the majority of the enriched proteins and metabolites were associated with 2,3-butanediol fermentation pathway (Figure 4d), and these proteins were rarely used in other alcohol-producing pathways. The 2,3-butanediol fermentation pathway is an oxidative pathway in glucose and glycerol metabolism, and the final product is 2,3-butanediol, with ethanol, acetate, and lactate as bypass products. The up-regulation of key enzymes including alcohol dehydrogenase, acetate kinase, acetolactate decarboxylase, diacetyl reductase, and acetoin reductase increases 2,3-butanediol and alcohol production. Thus, our data suggested that the *in vitro* ability of HiAlc *Kpn* to produce alcohol occurred *in vivo via* the 2,3-butanediol fermentation pathway under aerobic conditions (Figure 4d). These findings could be a promising clinical diagnosis marker for FLD induced by HiAlc *Kpn*.

### Effects of the 2,3-butanediol pathway in vitro

**Figure 5.**
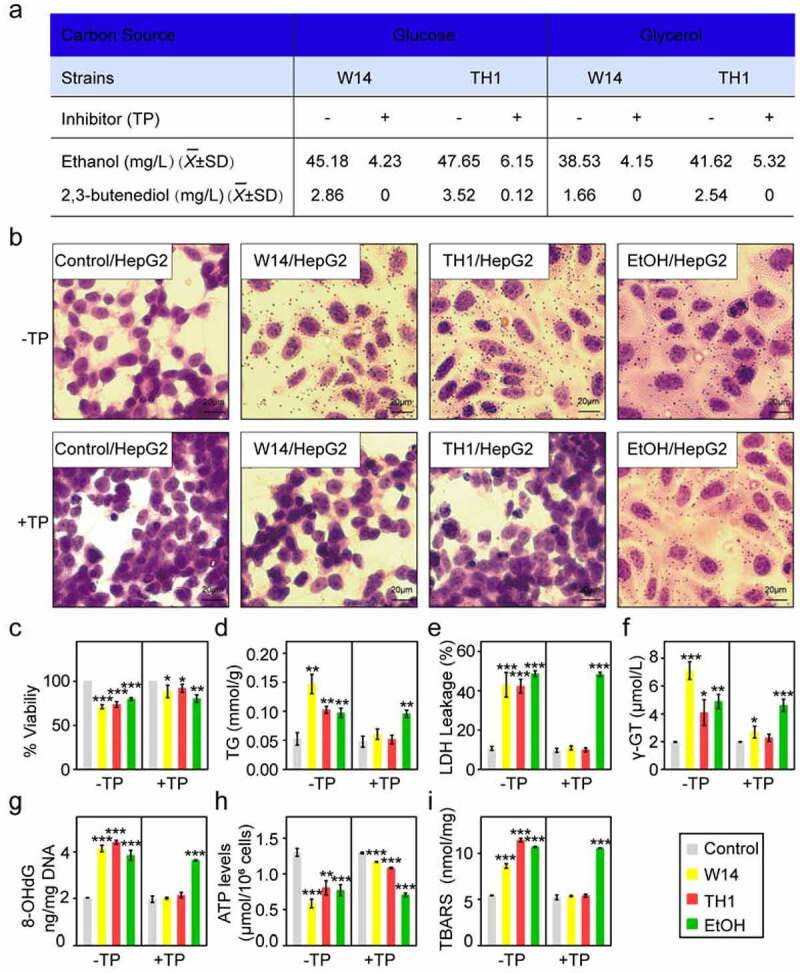
Hepatic steatosis caused by HiAlc *Kpn* with or without inhibitor in HepG2 cells. (a) Alcohol concentration of *Kpn* W14 and TH1 cells with or without inhibitor in the YPD medium wherein glycerol was used as the single carbon source. (b) Representative Oil Red O staining of HepG2 cells. Scale bar, 20 μm. (c-f) Percent viability (c), TG (mmol/g) (d), LDH leakage (%) (e), and γ-GT (µmol/L) (f) were used to assess injury and adipogenesis in HepG2 cells. (g-i) Oxidative mitochondrial DNA damage marker 8-OHdG levels (g), decreased ATP content in HepG2 cells (h), and increased cellular lipid hydroperoxide levels(i) were measured. (c-i) Data are expressed as mean ± SD of three independent experiments (n = 3). The *p* values were calculated by two-way ANOVA test

Acetolactate synthetase plays an important role in catalyzing the formation of two pyruvic acids into acetolactate and CO_2_, and then into acetoin *via* the 2,3-butanediol pathway. It is known that herbicides such as sulfonylurea (SU) and triazolopyrimidine (TP) are inhibitors of acetolactate synthetase. To determine whether ethanol was produced *via* the 2,3-butanediol pathway in HiAlc *Kpn*, TP was utilized to inhibit the yields of ethanol and 2,3-butanediol. The results showed that the alcohol concentration in HiAlc *Kpn* W14 or TH1 was decreased to below 10 mg/mL *in vitro* with increased TP (IC_50_ values were 42.6 µM to W14 and 43.7 µM to TH1, respectively). In addition, glycerol is non-fermentable by most microorganisms, with the exception of a group of bacteria including *Klebsiella, Clostridium, Enterobacter, Bacillus*, and *Lactobacillus* species.^10^ Of the strains capable of utilizing glycerol, *K. pneumoniae* was the first to be identified and has been reported to be one of the most competent organisms for producing high amounts of 2,3-butanediol, using glycerol as the sole carbon source *via* the 2,3-butanediol pathway. So, we cultivated HiAlc *Kpn* W14 and TH1 with glycerol as the sole carbon source. The strains showed similar ability for producing ethanol (38.5 g/L for W14 and 41.5 mg/L for TH1) and 2,3-butanediol (1.62 g/L for W14 and 2.54 mg/L for TH1) as those cultured with glucose as the sole carbon source (Figure 5a), suggesting that HiAlc *Kpn* can catalyze the conversion of glucose and glycerol to ethanol *via* the same metabolic pathway.

To further verify the effects of endo-ethanol produced by HiAlc *Kpn via* the 2,3-butanediol pathways on the development of steatosis, the hepatocarcinoma cell line HepG2 was incubated with the supernatant from cultured HiAlc *Kpn* W14 and TH1 in combination with TP (50 µM) for 72 h. The results showed that red fatty droplets, the contents of intracellular triglycerides, and the leakage of γ-GT into the medium increased dramatically in cells without TP treatment, while no morphological changes were observed in cells with TP treatment compared with the negative control (Figure 5b-i). These results indicated that HiAlc *Kpn* had the ability to induce typical morphological changes of steatosis in HepG2 cells *in vitro*.

### Confirmation of the involvement of 2,3-butanediol pathway in endogenous alcohol production

**Figure 6.**
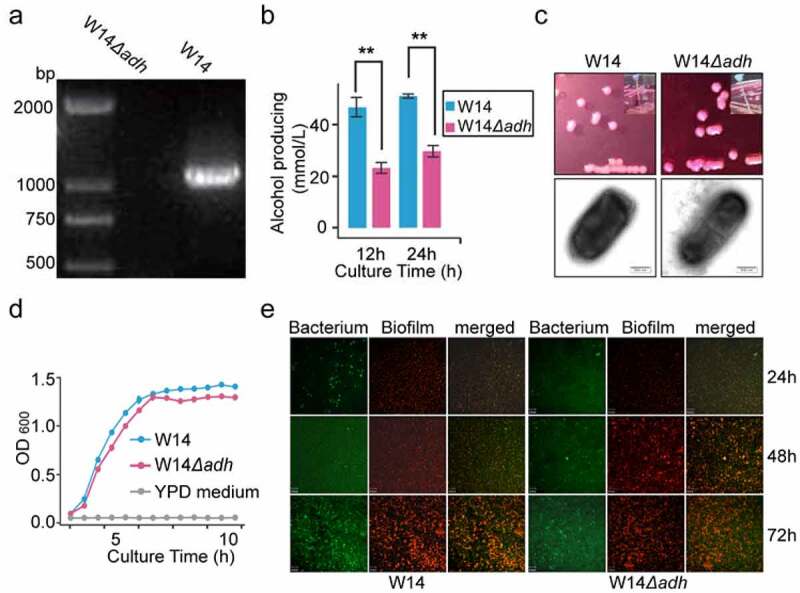
Determination of the pathway affecting the generation of alcohol by HiAlc *Kpn* W14 and W14*Δadh* knockout strains. (a) PCR amplification of the targeted region in the deletion mutant and wild-type bacteria. (b-d) Alcohol-producing abilities (b); images of typical colonies, wire drawing test, capsules (transmission electron microscopy), scale bar, 500 nm (c); and growth curves of HiAlc *Kpn* W14*Δadh* and W14 strains (d). Data are expressed as mean ± SD of ≥3 independent experiments (n = 3). The *p* values were calculated by two-way ANOVA test. (e) The images of biofilm formation by *K. pneumoniae* W14 and W14*Δadh* knockout strains. Scale bar, 29 μm

In our previous work, we demonstrated that HiAlc *Kpn* could be a causative agent of FLD.^4^ Alcohol dehydrogenase and acetolactate synthetase play important roles, catalyzing the reversible production of ethanol and 2,3-butanediol *via* the 2,3-butanediol pathway. To further confirm the involvement of the 2,3-butanediol pathway in endogenous alcohol production, we used the CRISPR-Cas9 system to knock out one of the *adh* genes: an essential gene encoding alcohol dehydrogenase in the 2,3- butanediol pathway in HiAlc *Kpn* W14 (Figure 6a). The results showed that the alcohol-producing ability of the bacteria was significantly reduced in the W14*Δadh* strain compared with the W14 strain, and similar to the results were observed in MedAlc *Kpn* (Figure 6b). The strain appeared to be a typical mucoid lactose fermenter, with a clear capsule and biofilm (Figure 6c-e). Furthermore, the growth rate of W14*Δadh* was similar to that of W14 (Figure 6d).

**Figure 7.**
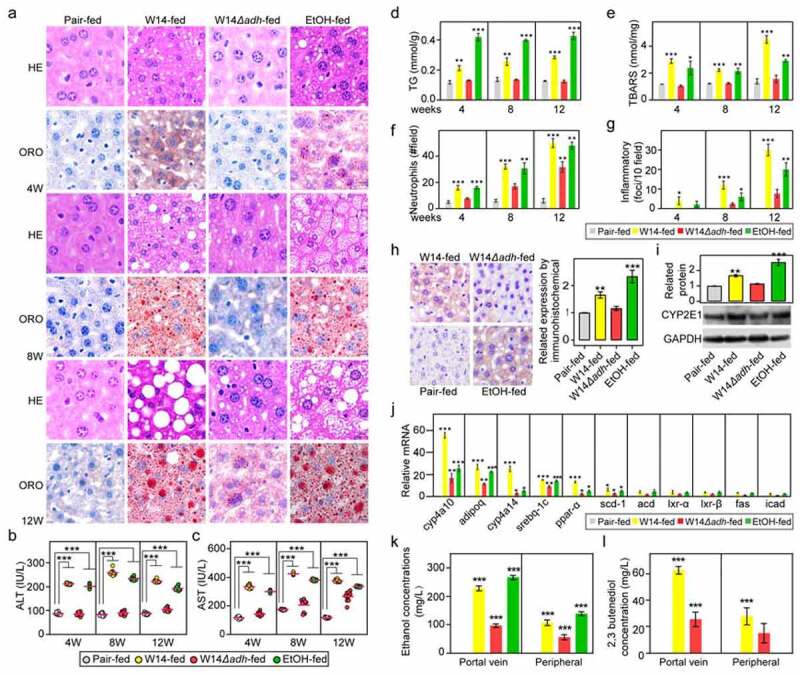
Effects of HiAlc *Kpn* mutant and wild-type strains on the induction of chronic hepatic steatosis. (a) Histological staining with H&E (40× magnification) and Oil Red O (40× magnification) of liver sections of SPF mice fed with HiAlc *Kpn* W14*Δadh* and W14 for 4, 8, and 12 weeks. Scale bar, 20 μm. (b-g) Liver injury induced by HiAlc *Kpn* W14*Δadh* in mice for 4, 8, and 12 weeks, which was assessed by the serum levels of ALT (b) (n = 10) and AST (c) (n = 10), and the contents of TG (d), TBARS (e), neutrophils (f), and inflammatory factors (g). (h, i) Hepatic expression of CYP2E1 in the liver tissues from HiAlc *Kpn*-fed mice, EtOH-fed mice, and pair-fed mice (4 weeks of gavage) by immunostaining (h) and Western blotting (i). (j) Real-time PCR shows the expression of genes related to fatty acid metabolism in the livers of mice after feeding with HiAlc *Kpn* W14*Δadh* and W14. (k, l) Comparison between the portal vein blood and peripheral blood, after feeding with the HiAlc *Kpn* W14 or W14*Δadh* to mice for 2 h; EtOH-fed mice and pair-fed mice were used as a control. (b-l) Data are expressed as mean ± SD of ≥3 independent experiments (n = 3). The *p* values were calculated by two-way ANOVA test

To explore the alcohol-producing ability of HiAlc bacteria in FLD *in vivo*, we also compared the effects of the wild-type and mutant of *Kpn* W14 strains on mice at the same concentrations and time points. Specific-pathogen-free (SPF) mice were fed with W14 (W14-fed group), W14*Δadh* (W14*Δadh*-fed group), ethanol (EtOH-fed group), or YPD medium (pair-fed group) for 4, 8, and 12 weeks. There were no significant differences in body weight or the liver-to-body mass ratio among the mice in any of the groups. Histological staining showed clear microsteatosis and macrosteatosis in the livers of the W14-fed and EtOH-fed mice at 4 and 8 weeks compared with that of the livers of the pair-fed mice (Figure 7a). However, in the W14*Δadh*-fed group, this damage did not occur until 12 weeks. Compared with the pair-fed mice, HiAlc *Kpn* W14-fed and ethanol-fed mice had significantly increased levels of aspartate transaminase (AST) and alanine transaminase (ALT) in the serum and increased levels of triglycerides (TG) and thiobarbituric acid-reactive substances (TBARS) in their livers (Figure 7b-e), which further indicated the existence of dynamic pathophysiological changes in the livers of these mice. In addition, the numbers of neutrophils and inflammatory foci also significantly increased over time after gavage (Figure 7f, g). Furthermore, the expression of the CYP2E1 protein (an essential internal control for ethanol exposure given that ethanol typically causes a robust increase in hepatic CYP2E1 expression) was also increased (Figure 7h, i), ten lipogenesis genes and PPARα (Figure 7j) were upregulated in the liver tissues of mice after gavage for 8 weeks. Correspondingly, compared with W14-fed and EtOH-fed mice, the W14*Δadh*-fed mice did not show apparent pathological changes at 4 weeks after feeding, although there were some steatotic hepatocytes observed at 12 weeks after feeding (Figure 7a), suggesting that the deletion of the *adh* gene significantly, even if not completely, reduced the ability of alcohol production.

To further demonstrate that ethanol is indeed produced by the microbiome, the ethanol and 2,3-butanediol concentrations in the portal vein blood and peripheral blood were compared. The results showed that the concentrations of ethanol and 2,3-butanediol were two times higher in the portal vein blood than in peripheral blood of colonized mice (Figure 7k, l). These results provide further evidence that the alcohol produced by the 2,3-butanediol pathway in *Kpn* is responsible for the steatosis effects on hepatocytes.

## Discussion

Under anaerobic conditions, organisms ranging from bacteria to mammalian cells excrete large quantities of fermentation products such as acetate, ethanol, or lactate. Strikingly, excretion of some fermentation products occurs widely even in the presence of oxygen in fast-growing bacteria and fungi, as well as mammalian (e.g., stem, immune, and cancerous) cells.^11–13^ This seemingly wasteful phenomenon, in which fermentation is used instead of the higher ATP-yielding respiration process for energy generation, is referred to as “overflow metabolism” (or the “Warburg effect” in the case of cancer). *K. pneumoniae* and *K. oxytoca* naturally produce high titers of 2,3-butanediol or 1,3-propanediol, and lactic acid as major fermentation end-products, along with relatively small amounts of byproducts (e.g., ethanol, acetoin, formic acid, acetic acid, and succinic acid) under micro-aerobic conditions.^14,15^ However, we showed that *K. pneumoniae* W14 and TH1 could elicit rapid growth rates and high alcohol production under anaerobic or aerobic conditions. These observations suggested that the overflow metabolism is probably a programmed global response used by cells to balance the conflicting proteomic demands of energy biogenesis and biomass synthesis for the rapid growth in *K. pneumoniae* W14 and TH1.

Recently, the importance of the gut-liver axis has become evident. The liver receives most of its blood supply from the intestine through the portal vein and is therefore one of the most exposed organs to potentially toxic factors originating in the gut, including all or part of the gut microbiota, and bioactive components of food that have been processed by the gut microbiota.^16^ Therefore, the well-characterized gut-liver axis may correctly be considered the gut-microbiota-liver network due to the high degree of interconnectedness between the microbiota and the host. Aided by high-throughput techniques and improved bioinformatics tools, it is possible to study the correlation between gene expression in the gut microbiota and host during FLD. It has also been proposed that the gut microbiota is an important environmental factor affecting host metabolism and that this is highly associated with FLD.^17–19^ However, there is variability between FLD patients with regard to the different metabolic pathways involved, and how the gut microbiota causes hepaticsteatosis remains unclear. Our previous work provided an extensive description of the key role of alcohol-producing *K. pneumoniae* in the pathogenesis of FLD, potentially offering a target for intervention or a clinical marker for the disease. Experimentally, *Kpn* with >20 mmol/L alcohol-producing ability could induce FLD in mice in less than 12 weeks. Our surveillance analysis of subjects with FLD indicated that a large proportion of FLD patients had endogenous alcohol possibly because of the presence of HiAlc *Kpn*. Herein, the current study, genome analysis of *K. pneumoniae* W14 and TH1 isolated from the intestinal microbiota of an ABS/NASH patient revealed that these HiAlc *Kpn* strains possessed 12 copies of ADHs, which play an important role in fermentation. That is, pyruvate resulting from glycolysis is converted to acetaldehyde and carbon dioxide, and then acetaldehyde is reduced to ethanol with the constant regeneration of nicotinamide adenine dinucleotide. *In vitro*, these strains could produce 83.7 mmol/L ethanol and 2,3-butanediol from glucose and fructose even under aerobic conditions. In addition, we unraveled through various omics data that the 2,3-butanediol pathway was involved in endogenous alcohol production during the liver disease.

Under normal conditions, ethanol is constantly produced by the intestinal microbiota in the human gut, as increased blood alcohol concentrations were detected after the intake of alcohol-free food,^20,21^ but the endogenous ethanol is rapidly and almost completely removed from portal blood by liver ADHs, catalases, and the microsomal ethanol-oxidizing system. It remained to be determined whether HiAlc *Kpn* utilizes the 2,3-butanediol fermentation pathway to produce high-level endogenous alcohol *in vivo*, which would be different from the alcohol-production pathway used by yeast. Considering all of the potential alcohol-producing pathways in bacteria, we found that the majority of the enriched proteins and metabolites were associated with the 2,3-butanediol fermentation pathway (Figure 4d), which is a neglected pathway for alcohol production from glucose and glycerol metabolism *in vivo*. Accordingly, the key upregulated enzymes and metabolites all belonged to this pathway (Figure 4d). On the one hand, the pathway is capable of efficiently transforming sugar and glycerol into alcohols and acids. On the other hand, the metabolites derived from alcohol catabolism, including acetaldehyde, acetic acid and fatty acid ethyl esters, may also cause tissue injury and hepatic steatosis.

Normally, ethanol production dominates in *Saccharomyces cerevisiae* sugar metabolism. 2,3-Butanediol is a high-value chemical usually produced petrochemically but can also be synthesized by some bacteria, such as *Enterobacter, Klebsiella, Serratia*, and *Bacillus*. Two fermentation types exist in the *Enterobacteriaceae* family: mixed-acid fermenters that produce substantial amounts of lactate, formate, acetate, and succinate, resulting in lethal medium acidification, and 2,3-butanediol fermenters that switch to the production of the neutral compounds acetoin and 2,3-butanediol and even deacidify the environment after an initial acidification phase, thereby avoiding cell death. The synthesis of 2,3-butanediol from pyruvate requires three steps. First, the conversion of two molecules of pyruvate to α-acetolactate is catalyzed by the α-acetolactate synthase (α-ALS). Second, α-acetolactate is decarboxylated to acetoin by the α-acetolactate decarboxylase (α-ALD). Third, acetoin is reduced to 2,3-butanediol by 2,3-butanediol dehydrogenase (BDH), which can also catalyze the reverse reaction.

Currently, gut microbiome as a noninvasive biomarker for the prevention, diagnosis, treatment, and drug response in FLD is concerned.^22^ Fecal markers for diagnosis and prevention have been proposed in some diseases such as some colorectal cancer and breast cancer subtypes.^23,24^ Therefore, high-throughput sequencing of HiAlc *Kpn* in fecal may help diagnose or predict the mortality risk in FLD. A growing number of studies have demonstrated that microbiota metabolites, such as short chain fatty acids, bile acids, virulence factors, ethanol, and others, are responsible for FLD pathophysiology.^25^ Here, we showed that HiAlc *Kpn* is a potential causative agent of FLD *via* endogenous ethanol production, which is mediated by the 2,3-butanediol pathway. This study shed a new insight on the future diagnosis, prevention, and therapeutics of FLD. First, carbohydrate is the substrate of alcohol production for HiAlc *Kpn*. Accordingly, we can stage FLD or its progressive form by determining the relationship between the carbohydrate consumption and the amounts of endogenous ethanol production. In addition, we can explore the small molecular inhibitor of HiAlc *Kpn* metabolic enzymes that targets the 2,3-butanediol pathway to block the endogenous ethanol production. Furthermore, tackling FLD and its related metabolic complications by promoting the conversion of endogenous ethanol to useful metabolites or promoting excretion from the organism.

*K. pneumoniae* is a potentially useful producer in the industry because of its wide substrate spectrum, high efficiency, and cultural adaptability.^15,26^ It is able to convert all the major sugars present in hemicellulose and cellulose hydrolyzates into 2,3-butanediol.^27^ This ethanol biosynthetic pathway needs to be metabolically replaced with a 2,3-butanediol producing pathway to produce 2,3-butanediol efficiently. The production of 2,3-butanediol was dramatically improved with the inactivation of *adh*E and *pta*. Furthermore, the inactivation of *ldh*A promotes cell growth and a shorter fermentation time. A double mutant strain with the deletion of *adh*E and *ldh*A resulted in accelerated fermentation and higher 2,3-butanediol production.^28^ Further investigations are needed to confirm whether HiAlc *Kpn* uses the 2,3-butanediol pathway for endogenous alcohol production. The novelties of the present study are that we discovered the use of the 2,3-butanediol fermentation pathway within the intestine and we also determined the fermentation pathway of alcohol production. Intriguingly, our study revealed potential connections between cryptic ABS and FLD, although further evidence is needed to confirm this.

## Limitations of study

The key limitation of this study is the lack of a cohort of ABS patients to prove the effect of 2,3-butanediol fermentation pathway. Also, future studies should pay attention to the effects of other metabolites produced from 2,3-butanediol fermentation and catabolism. Moreover, the pathways for generating endogenous alcohol within the intestine remain to be investigated, and the balance of endogenous alcohol production and conversion *in vivo* also needs to be clarified.

## Materials and methods

### Reagents and resources

The whole genome sequence of *K. pneumoniae* W14 can be found on https://www.ncbi.nlm.nih.gov/nuccore/NZ_CP015753.1; the whole genome sequence of *K. pneumoniae* TH1 can be found on https://www.ncbi.nlm.nih.gov/nuccore/NZ_CP016159.1; Further, Affymatrix Microarray Gene Expression Analysis data can be found on https://www.ncbi.nlm.nih.gov/geo/query/acc.cgi?acc=GSE102489. The raw reads for 16S rDNA and whole metagenome data for all samples from the patient and mice can be found in the NCBI Sequence Read Archive under accession numbers SRR5934751 and SRR5934662. CYP2E1 antibody (Abcam, ab28146), SYBR Premix Ex Taq II (TaKaRa, RR820A), 2-D Clean-Up Kit (GE Healthcare, 80–6484-51), 2-D Quant Kit (GE Healthcare, 80–6483-56), and QIAamp DNA Stool Mini Kit (QIAamp, 51504).

### Specimens and patients

A 27-year-old Chinese male presented with a rare case of nonalcoholic steatohepatitis (NASH) accompanied with bacterial auto-brewery syndrome (ABS), after eating carbohydrates, who became inebriated with ethanol concentrations ≥190 mg/dL, even reaching ~ 400 mg/dL in his blood; he was believed to be a “closet drinker”. However, the underlying cause of ABS was later thought to be an overgrowth of HiAlc *Kpn* that ferments carbohydrates into alcohol in the gut.^4^

Using YPD medium supplemented with 5% alcohol and under aerobic or anaerobic culture conditions, high and medium alcohol-producing strains were isolated from fecal samples of the NASH/ABS patient at a morbid stage. A number of techniques including PCR amplification and sequencing of 16S rDNA, microbial morphological analysis, electron microscopy, MALDI-TOF mass spectrometry, and automated microdilution were performed to identify all bacterial colonies from the isolates, and eight *Kpn* strains were collected. The standard strain *Kpn* ATCC BAA-2146 was used as a control. A longitude cohort of FLD participants was performed as previously described.^4^

### Mice and rabbits

Japanese white male rabbits (n = 6, 28–32 weeks old) were provided and kept by the Experimental Animal Center at the Academy of Military Medical Sciences. The mean body weight of the rabbits was 3,549 g (±203 g). Specific-pathogen-free (SPF) C57BL/6 J male mice were obtained and maintained at the Academy of Military Medical Sciences in accordance with Academy of Military Medical Sciences Animal Resource Center and the Institutional Animal Care and Use Committee (IACUC) guidelines.

### Bacterial strains and growth conditions

The trains were cultivated and purified in both YPD and MacConkey medium (with or without 5% alcohol) under anaerobic and aerobic conditions at 37°C for 24 h. Anaerobic conditions were achieved in jars using AnaeroPacks (Mitsubishi Gas Chemical Company, Tokyo, Japan).

The knock-out of the *adh* gene in HiAlc *Kpn* W14 (1038 bp, site 202066–203103 in genome) was performed using the CRISPR-Cas9 system. A protospacer adjacent motif (PAM) was designed. The sequences of *adh* primers were F-ATGAAGTATGTGAATCTGGG and R-TTAATAGTTCTGGATCGCTG. The knock-out strain was named as W14*Δadh* mutant.

### Genome sequencing of HiAlc and MedAlc *Kpn* strains

The eight *K. pneumoniae* strains (W14, TH1, LA13.1, LA4.1, F4.1, F1.1, F3.1, and TE2.1) were sequenced by PacBio and Illumina Hiseq. The SMRT Link software package was used for *de novo* assembly of third-generation sequencing data. The genome sequence of *K. pneumoniae* ATCC BAA-2146 or *K. pneumoniae* (strain ATCC 700721/MGH 78578) was used as a reference. The protein-coding genes were predicted using Prokka 1.12 according to the manual. Functional annotations were added by comparing protein sequences to the public databases. The COG assignment was performed by the program RPS-BLAST at an e-value cutoff of 1e-2; the best hit was selected when the alignment length reached at least 70% of the length of the query gene and subject gene. Protein sequences were compared with those of KEGG genes using USEARCH with an e-value cutoff of 1e-5. The top five hits were selected when the sequence identity was greater than or equal to 30%, and the alignment length was at least 70% of the length of the query gene and subject gene.^29^

### Biological characteristics of HiAlc and MedAlc *Kpn*

Transmission electron microscopy and the identification of lactose fermenters on MacConkey agar were used to observe the capsules of HiAlc and MedAlc *Kpn* strains. Moreover, confocal microscopy was used to identify biofilm formation by HiAlc and MedAlc *Kpn* strains that were diluted to 1:50 in YPD medium and cultivated in six-well plates for 24, 48, and 72 h.

In addition, according to the sequences of the *galF* and *gnd* genes, the K antigen type of the strains was determined. Internal fragments of seven house-keeping genes (*rpo*B, *gap*A, *mdh, pgi, pho*E, *inf*B, and *ton*B) were amplified using the following steps: pre-denaturation of the reaction mixture for 2 min at 94°C; 35 cycles of 20 s at 94°C, 30 s at an appropriate annealing temperature (50°C/60°C /45°C), and 30 s at 72°C; and a final elongation step for 5 min at 72°C. The amplicons were sequenced by Sangon Biotec Co., Ltd., Shanghai, China. The allele number for each gene was assigned on the basis of the information in the *Klebsiella pneumoniae* MLST database (http://pubmlst.org/Klebsiella/). A combination of the allelic sequences of the seven genes yielded the allelic profile.

Eighteen potential virulence-related factors identified from the literature and databases were measured in HiAlc and MedAlc *Kpn* strains, as well as the standard strain ATCC BAA-2146 (LowAlc). Antibiotic susceptibility testing was conducted by disk diffusion in accordance with the guidelines of the Clinical and Laboratory Standards Institute and was confirmed using the Vitek 2 System with antibiotics. The standard strain *Kpn* ATCC BAA-2146 was used as a control.

### Measurement of alcohol concentration

The alcohol concentrations of all in the strains and fecal samples were measured using a commercial ethanol assay kit (BioVision, Milpitas, CA, USA), following the manufacturer’s instructions. The blood samples from the mice gavaged with HiAlc *Kpn* were analyzed by the headspace gas chromatography method (HS-GC, 6850 Agilent, with a flame ionization detector FID-Headspace).^30^ The alcohol-producing abilities of HiAlc and MedAlc *Kpn* were measured using YPD medium containing 2%, 4%, 6%, 8%, and 10% fructose or glucose as the sole carbon source under aerobic conditions, and the growth curves of HiAlc and MedAlc *Kpn* were determined.

### Metabolome analysis by GC-MS

Metabolome analysis was performed on fresh fecal samples from FLD mice induced by HiAlc *Kpn* and the cultures of HiAlc *Kpn*, which were preconditioned according to the manufacturer’s instructions. GC-TOF/MS analysis was performed using the LECO Pegasus 4D system (Leco Corporation, St Joseph, MI, USA). The inlet temperature was 250°C. The carrier gas was helium, which was kept at a constant flow rate of 1.0 mL/min. The GC temperature programming was set to 1 min isothermal heating at 70°C, followed by ramping of the temperature at 5°C/min up to 280°C, where the temperature was held for 10 min. The transfer line and ion-source temperatures were 250°C and 220°C, respectively. Electron impact ionization (70 eV) was set at a detector voltage of 1,575 V. Ten scans per second were recorded over the full mass range of 50–800 m/z. Chromatogram acquisition, library research, and peak area calculation were performed using ChromaTOF software (Version 4.5, LecoCorp.). Significantly different molecules were selected by their FDR-adjusted *P* values.

### Proteomics analysis of HiAlc *Kpn* bacteria in vivo and in vitro

An *in vivo/in vitro* culture assay using a rabbit intestinal model was performed as described previously.^31^ Here, 20 mL of bacterial suspension was placed in dialysis tubing with a molecular weight cutoff of 20,000 Da. After the rabbits were anesthetized, the HiAlc *Kpn* culture was implanted aseptically within the colon through a 1 cm incision, and the incision was then closed using surgical staples. The tubing containing the HiAlc *Kpn* culture was either incubated in the rabbit intestine for four hours or in vitro at 37°C. The rabbit was generally ambulatory within 4 h. Subsequently, the dialysis bag containing the HiAlc *Kpn* culture was taken out and HiAlc *Kpn* was harvested for alcohol determination and proteomics analysis. The experiment was performed at least six times.

Whole cellular protein extracts of HiAlc *Kpn* W14, TH1, and ATCC BAA-2146 were isolated by 2D gel electrophoresis and MALDI-TOF/TOF MS/MS was performed on a Bruker Ultraflex III TOF/TOF-MS (Bruker Daltonics GmbH, Bremen, Germany) to identify the proteins. The proteins were considered differentially expressed if their relative intensity differed more than three-fold between the two conditions. Each experiment was performed at least three times.

### Construction of FLD mouse model with strain gavage

C57BL/6 J SPF mice were fed with a chow diet, 60 kcal % fat (Research Diets, New Brunswick, NJ) for 5 days. The mice were then randomly divided into four groups and gavaged once every two days with 200 μL of ~10^7^ CFU of *K. pneumoniae* for 4, 8, or 12 weeks: HiAlc *Kpn* W14-fed and W14*Δadh*-fed groups were gavaged a single dose of *K. pneumoniae* strains suspended in YPD medium (~10^7^ CFU, 200 μL); ethanol groups as the positive control were gavaged a single dose of ethanol (40% ethanol, 200 μL); the pair-fed group as the negative control were fed with the YPD medium (200 μL). The gavage was always performed in the early morning. In this case, 100% mice survived after feeding with the bacterial strain or ethanol. The mice were euthanized. The number of animals for each subpanel was ≥ 6, while the number of experiments was ≥ 3.

### Histological and physiological assays in mice

The body weights of the mice were recorded weekly. Fresh feces of mice were collected at 6, 8, and 12 weeks after feeding for metabolomic analyses. The serum, liver and tissues were collected from the dissected mice. Subsequently, the harvested livers were fixed in 10% formalin and processed *via* H&E and Oil Red O staining. Detecting the serum indices including ALT, AST, TG, TBARS, and neutrophils, and inflammatory foci in the liver tissues were used to assess liver injury. Quantification of neutrophils and inflammatory foci (microscopically, 50 field per section) was performed, and calculated the average.

To detect the hepatic expression of genes related to lipogenesis, specific primers targeting the genes of interest were used in real-time qPCR. Briefly, quantitative PCR was performed using a Light Cycler 2.0 PCR sequence detection system and the Fast Start DNA Master SYBR Green kit. Melting-point-determination analysis was used to confirm the specificity of the amplification products. The copy numbers of target genes from each sample were calculated by comparing the Ct values obtained from the standard curves with the Light Cycler 4.0 software. The standard curves were created using a serial 10-fold dilution of DNA from pure cultures, corresponding to 10^1^–10^10^ copies/g feces. The data are expressed as the mean values of duplicate real-time qPCR analyses.

### Immunostaining and Western blot analysis

The hepatic expression of CYP2E1 in the liver tissues from HiAlc *Kpn* W14-fed, W14*Δadh*-fed, EtOH-fed, and pair-fed mice after 4 weeks was analyzed by immunostaining and Western blotting. The proteins of liver tissues or cells were size-separated by 10% SDS-PAGE and, transferred to PVDF membranes. The membranes were blocked with 5% nonfat dry milk before being incubated with primary antibodies against CYP2E1/FITC. After incubating with secondary antibodies against mouse IgG or rabbit IgG, the membranes were visualized using ECL-Plus chemiluminescence reagent. In addition, immunohistochemical analysis with anti-CYP2E1/FITC antibody was performed to detect the expression of CYP2E1 in the liver cells of mice. Data are expressed as the mean (±SD) of at least three independent experiments.

### Ethanol concentrations in portal vein blood and peripheral blood of mice

After feeding with HiAlc *Kpn* W14 or W14*Δadh* for 2 h, the portal vein blood and peripheral blood of mice were collected. GC-MS was used to measure the ethanol and 2,3- butanediol concentrations in the mouse blood samples. The pair-fed mice were used as negative control, while the ethanol group fed a single dose of ethanol (40% ethanol, 200 μL) was used as a positive control.

### Statistical analysis

Statistical analysis was performed using the SPSS 22.0 software. Data are expressed as means ± standard deviations (SD). Statistical analysis was carried out using two-way ANOVA to compare multiple groups or the student’s *t* test to compare two groups. A *p* value <.05 was considered statistically significant.

## Data Availability

The whole genome sequences of *K. pneumoniae* W14 and TH1 have been deposited in the GenBank database under accession numbers NZ_CP015753.1 NZ_CP016159.1 for W14 and TH1, respectively.

## Abbreviations

YPD, yeast extract peptone dextrose; ADH, alcohol aldehyde dehydrogenase; ALD, acetaldehyde dehydrogenase; AR, aldose reductase; GC-MS, gas chromatography mass spectrometry; HepG2, hcuman hepatocellular carcinomas; TG, triglyceride; LDH, lactate dehydrogenase; γ-GT, γ-glutamyl transpeptidase; TBARS, thiobarbituric acid reactive substance.

## References

[1] Grundmann H, Glasner C, Albiger B, Aanensen DM, Tomlinson CT, Andrasevic AT, Canton R, Carmeli Y, Friedrich AW, Giske CG, et al. Occurrence of carbapenemase-producing Klebsiella pneumoniae and Escherichia coli in the European survey of carbapenemase-producing Enterobacteriaceae (EuSCAPE): a prospective, multinational study. Lancet Infect Dis. 2017;17(2):153–18. doi:10.1016/S1473-3099(16)30257-2. 27866944

[2] Dubinkina VB, Tyakht AV, Odintsova VY, Yarygin KS, Kovarsky BA, Pavlenko AV, Ischenko DS, Popenko AS, Alexeev DG, Taraskina AY, et al. Links of gut microbiota composition with alcohol dependence syndrome and alcoholic liver disease. Microbiome. 2017;5(1):141. doi:10.1186/s40168-017-0359-2. 29041989 PMC5645934

[3] Lanzke N, Kleinwachter R, Kerschischnik S, Sargsyan L, Groneberg DA, Kamradt T, Liesenfeld O, Krenn V, Sander M, Spies C. Differential effects of ethanol on IFN-gamma- and TNF-alpha-producing splenic T lymphocytes in a murine model of gram-negative pneumonia. Addict Biol. 2007;12(1):59–68. doi:10.1111/j.1369-1600.2006.00042.x. 17407498

[4] Yuan J, Chen C, Cui J, Lu J, Yan C, Wei X, Zhao X, Li N, Li S, Xue G, et al. Fatty liver disease caused by high-alcohol-producing Klebsiella pneumoniae. Cell Metab. 2019;30(4):675–688. doi:10.1016/j.cmet.2019.08.018. 31543403

[5] Gronenberg LS, Marcheschi RJ, Liao JC. Next generation biofuel engineering in prokaryotes. Curr Opin Chem Biol. 2013;17(3):462–471. doi:10.1016/j.cbpa.2013.03.037. 23623045 PMC4211605

[6] Hsieh PF, Lu YR, Lin TL, Lai LY, Wang JT. Klebsiella pneumoniae type VI secretion system contributes to bacterial competition, cell invasion, Type-1 fimbriae expression, and in vivo colonization. J Infect Dis. 2019;219(4):637–647. doi:10.1093/infdis/jiy534. 30202982 PMC6350951

[7] Lin TL, Lee CZ, Hsieh PF, Tsai SF, Wang JT. Characterization of integrative and conjugative element ICEKp1-associated genomic heterogeneity in a Klebsiella pneumoniae strain isolated from a primary liver abscess. J Bacteriol. 2008;190(2):515–526. doi:10.1128/JB.01219-07. 17981959 PMC2223707

[8] Lawlor MS, O’Connor C, Miller VL. Yersiniabactin is a virulence factor for Klebsiella pneumoniae during pulmonary infection. Infect Immun. 2007;75(3):1463–1472. doi:10.1128/IAI.00372-06. 17220312 PMC1828572

[9] Lawley TD, Klimke WA, Gubbins MJ, Frost LS. F factor conjugation is a true type IV secretion system. Fems Microbiol Lett. 2003;224(1):1–15. doi:10.1016/S0378-1097(03)00430-0. 12855161

[10] Oh BR, Lee SM, Heo SY, Seo JW, Kim CH. Efficient production of 1,3-propanediol from crude glycerol by repeated fed-batch fermentation strategy of a lactate and 2,3-butanediol deficient mutant of Klebsiella pneumoniae. Microb Cell Fact. 2018;17(1):92. doi:10.1186/s12934-018-0921-z. 29907119 PMC6003044

[11] Basan M, Hui S, Okano H, Zhang Z, Shen Y, Williamson JR, Hwa T. Overflow metabolism in Escherichia coli results from efficient proteome allocation. Nature. 2015;528(7580):99–104. doi:10.1038/nature15765. 26632588 PMC4843128

[12] Hanahan D, Weinberg RA. Hallmarks of cancer: the next generation. Cell. 2011;144(5):646–674. doi:10.1016/j.cell.2011.02.013. 21376230

[13] Vander HM, Cantley LC, Thompson CB. Understanding the Warburg effect: the metabolic requirements of cell proliferation. Science. 2009;324(5930):1029–1033. doi:10.1126/science.1160809. 19460998 PMC2849637

[14] Zhang G, Yang G, Wang X, Guo Q, Li Y, Li J. Influence of blocking of 2,3-butanediol pathway on glycerol metabolism for 1,3-propanediol production by Klebsiella oxytoca. Appl Biochem Biotechnol. 2012;168(1):116–128. doi:10.1007/s12010-011-9363-3. 21915590

[15] Ma C, Wang A, Qin J, Li L, Ai X, Jiang T, Tang H, Xu P. Enhanced 2,3-butanediol production by Klebsiella pneumoniae SDM. Appl Microbiol Biotechnol. 2009;82(1):49–57. doi:10.1007/s00253-008-1732-7. 18949476

[16] Llorente C, Schnabl B. The gut microbiota and liver disease. Cell Mol Gastroenterol Hepatol. 2015;1(3):275–284. doi:10.1016/j.jcmgh.2015.04.003. 26090511 PMC4467911

[17] Del CF, Nobili V, Vernocchi P, Russo A, De Stefanis C, Gnani D, Furlanello C, Zandona A, Paci P, Capuani G, et al. Gut microbiota profiling of pediatric nonalcoholic fatty liver disease and obese patients unveiled by an integrated meta-omics-based approach. Hepatology. 2017;65(2):451–464. doi:10.1002/hep.28572. 27028797

[18] Leung C, Rivera L, Furness JB, Angus PW. The role of the gut microbiota in FLD. Nat Rev Gastroenterol Hepatol. 2016;13(7):412–425. doi:10.1038/nrgastro.2016.85. 27273168

[19] Zhu L, Baker SS, Gill C, Liu W, Alkhouri R, Baker RD, Gill SR. Characterization of gut microbiomes in nonalcoholic steatohepatitis (NASH) patients: a connection between endogenous alcohol and NASH. Hepatology. 2013;57(2):601–609. doi:10.1002/hep.26093. 23055155

[20] Musso G, Gambino R, Cassader M. Non-alcoholic fatty liver disease from pathogenesis to management: an update. Obes Rev. 2010;11(6):430–445. doi:10.1111/j.1467-789X.2009.00657.x. 19845871

[21] Cope K, Risby T, Diehl AM. Increased gastrointestinal ethanol production in obese mice: implications for fatty liver disease pathogenesis. Gastroenterology. 2000;119(5):1340–1347. doi:10.1053/gast.2000.19267. 11054393

[22] Sharpton SR, Schnabl B, Knight R, Current Concepts LR. Opportunities, and challenges of gut microbiome-based personalized medicine in nonalcoholic fatty liver disease. Cell Metab. 2021;33(1):21–32. doi:10.1016/j.cmet.2020.11.010. 33296678 PMC8414992

[23] Yu J, Feng Q, Wong SH, Zhang D, Liang QY, Qin Y, Tang L, Zhao H, Stenvang J, Li Y, et al. Metagenomic analysis of faecal microbiome as a tool towards targeted non-invasive biomarkers for colorectal cancer. Gut. 2017;66(1):70–78. doi:10.1136/gutjnl-2015-309800. 26408641

[24] Banerjee S, Tian T, Wei Z, Shih N, Feldman MD, Peck KN, DeMichele AM, Alwine JC, Robertson ES. Distinct microbial signatures associated with different breast cancer types. Front Microbiol. 2018;9:951. doi:10.3389/fmicb.2018.00951. 29867857 PMC5962706

[25] Canfora EE, Meex R, Venema K, Blaak EE. Gut microbial metabolites in obesity, NAFLD and T2DM. Nat Rev Endocrinol. 2019;15(5):261–273. doi:10.1038/s41574-019-0156-z. 30670819

[26] Petrov K, Petrova P. High production of 2,3-butanediol from glycerol by Klebsiella pneumoniae G31. Appl Microbiol Biotechnol. 2009;84(4):659–665. doi:10.1007/s00253-009-2004-x. 19396438

[27] Jung MY, Mazumdar S, Shin SH, Yang KS, Lee J, Oh MK. Improvement of 2,3-butanediol yield in Klebsiella pneumoniae by deletion of the pyruvate formate-lyase gene. Appl Environ Microbiol. 2014;80(19):6195–6203. doi:10.1128/AEM.02069-14. 25085487 PMC4178695

[28] Guo X, Cao C, Wang Y, Li C, Wu M, Chen Y, Zhang C, Pei H, Xiao D. Effect of the inactivation of lactate dehydrogenase, ethanol dehydrogenase, and phosphotransacetylase on 2,3-butanediol production in Klebsiella pneumoniae strain. Biotechnol Biofuels. 2014;7(1):44. doi:10.1186/1754-6834-7-44. 24669952 PMC3974439

[29] Huntemann M, Ivanova NN, Mavromatis K, Tripp HJ, Paez-Espino D, Palaniappan K, Szeto E, Pillay M, Chen IM, Pati A, et al. Erratum to: the standard operating procedure of the DOE-JGI microbial genome annotation pipeline (MGAP v.4). Stand Genomic Sci. 2016;11(27). doi:10.1186/s40793-016-0148-8 PMC4800771 27004084

[30] Watanabe-Suzuki K, Seno H, Ishii A, Kumazawa T, Suzuki O. Ultra-sensitive method for determination of ethanol in whole blood by headspace capillary gas chromatography with cryogenic oven trapping. J Chromatogr B Biomed Sci Appl. 1999;727(1–2):89–94. doi:10.1016/s0378-4347(99)00063-8. 10360426

[31] Yuan J, Wang B, Sun Z, Bo X, Yuan X, He X, Zhao H, Du X, Wang F, Jiang Z, et al. Analysis of host-inducing proteome changes in bifidobacterium longum NCC2705 grown in Vivo. J Proteome Res. 2008;7(1):375–385. doi:10.1021/pr0704940. 18027903

